# Mechanisms of exercise intervention in type 2 diabetes: a bibliometric and visualization analysis based on CiteSpace

**DOI:** 10.3389/fendo.2024.1401342

**Published:** 2024-08-01

**Authors:** Yue Jin, Kang Wan, Cheng Liu, Wei Cheng, Ru Wang

**Affiliations:** ^1^ School of Exercise and Health, Shanghai University of Sport, Shanghai, China; ^2^ Physical Education College, Henan Sport University, Zhengzhou, China; ^3^ Department of Endocrinology, Yangpu Hospital, School of Medicine, Tongji University, Shanghai, China

**Keywords:** type 2 diabetes, exercise intervention, mechanisms, bibliometric analysis, CiteSpace

## Abstract

**Objective:**

Type 2 diabetes (T2D) is a common chronic metabolic disease, and its prevalence is increasing globally. Exercise is crucial for T2D management, yet many aspects of its mechanisms remain unclear. This study employs CiteSpace to reveal research hotspots and frontier issues in exercise intervention for T2D.

**Method:**

A literature review spanning from January 1, 2013 to December 31, 2022, was conducted using the Web of Science Core Collection (WoSCC), with keywords including “exercise,” “type 2 diabetes,” and “mechanisms.” We analyzed network diagrams generated by CiteSpace, which depicted relationships among countries, authors, and keywords.

**Results:**

This study includes 1,210 English papers from 555 journals, affiliated with 348 institutions across 80 countries/regions. Notably, the United States, China, and the United Kingdom account for nearly half of all publications. The University of Copenhagen leads in publication volume, followed by Harvard Medical School and the University of Colorado. Key authors include Kirwan, John P (Case Western Reserve University), Malin, Steven K (Rutgers University), and Pedersen, Bente Klarlund (University of Copenhagen). Based on co-occurrence analysis of keywords, it is evident that terms such as “disease,” “glucagon-like peptide 1,” and “cardiovascular risk factor” exhibit high intermediary centrality.

**Conclusion:**

The analysis highlights ongoing investigations into molecular mechanisms, such as β-cell function enhancement, exerkines, and epigenetic mechanisms. Emerging areas include exercise response heterogeneity, circadian rhythm regulation, transcription factors, neurotrophic factors, and mitochondrial function. Future studies should prioritize understanding interactions between different exercise mechanisms and optimizing exercise prescriptions for T2D. Exercise prescriptions are crucial for effective interventions. Collaboration between countries and institutions is essential to understand the influences of different genetic backgrounds and environmental factors. Currently, a combination of aerobic and resistance training is considered the optimal form of exercise. However, considering time efficiency, high-intensity interval training (HIIT) has gained widespread attention and research due to its ability to achieve similar exercise effects in a shorter duration. Additionally, circadian rhythm regulation may affect the exercise outcomes of diabetic individuals at different times of the day, particularly concerning the specific types, doses, and intensities used for precision intervention in T2D.

## 1 Introduction

Diabetes, a metabolic disorder characterized by elevated blood glucose levels, is primarily attributed to the decline in functional β-cell mass (1). Global Burden of Diseases, Injuries, and Risk Factors Study (GBD) 2021 Diabetes Collaborators anticipate that by the year 2050, the global prevalence of diabetes will affect over 1.31 billion people, with over 90% diagnosed with T2D (2). The adverse effects of T2D include both short-term and long-term complications, significantly diminishing patients’ quality of life and increasing the susceptibility to other health issues and mortality (3). Individuals with T2D have a substantially heightened predisposition to cardiovascular diseases, exposing them to increased risks of coronary artery disease, myocardial infarction, heart failure, and stroke (4). Prolonged elevation of glycemic may contribute to renal dysfunction, potentially necessitating interventions such as dialysis or kidney transplantation (5, 6). T2D can also result in retinopathy, leading to visual impairment or, in severe cases, complete blindness (7). Furthermore, high blood glucose incites neuropathic damage, particularly affecting the extremities and resulting in numbness, pain, or weakness in the hands and feet (8, 9). The intricate interplay of these multi-faceted complications seriously impacts the physiological systems of people with T2D.

Researchers have established regular exercise as an effective strategy for managing individuals with T2D (10). Aerobic training can reduce hemoglobin A1c (HbA1c) levels, triglycerides, blood pressure, and insulin resistance among individuals with T2D (11–13). The reduction in HbA1c levels in people with T2D is associated with a decreased risk of CVD (14). Study shows that for every 1% decrease in HbA1c levels, microvascular complications decrease by 37% and myocardial infarctions decrease by 14% (15). Additionally, changes in muscle metabolism are crucial in the development and prevention of numerous chronic diseases (16). By increasing muscle mass, resistance exercise facilitates glucose uptake by muscles from the bloodstream, decreasing blood glucose levels both during and after exercise (17–19). Growing evidence shows that combined aerobic and resistance exercise is more effective for glycemic control than either aerobic or resistance exercise alone (20–23). Exercise further plays a vital role in weight reduction and maintenance, a critical aspect for people with T2D (24). The Action for Health in Diabetes (Look AHEAD) study shows that in overweight or obese adults with type 2 diabetes, intensive lifestyle interventions aimed at weight loss can improve the control of cardiovascular risk factors, reduce obesity-related complications, and enhance patients’ quality of life, although they do not decrease the incidence of cardiovascular events (25). Given the continuous rise in the incidence of T2D and the escalating emphasis on exercise intervention, the volume of related literature is increasing. A large body of research is delving into the biological mechanisms of exercise on metabolism (26, 27). Consequently, a retrospective analysis of published studies on the mechanisms of exercise in improving T2D is imperative. A systematic review of the advancements in this domain can aid researchers in identifying current issues, offering insights for the future progression.

Bibliometrics, as a branch of informatics, entails the quantitative analysis of patterns in scientific literature to discern emerging trends and knowledge structures within research fields (28–30). It serves as an objective tool for scrutinizing the current status of disciplines and reflecting the development of a subject (31). CiteSpace is a visual analytics tool explicitly designed to assist researchers in exploring the structure and evolutionary trends of scientific literature data (32). Developed by Professor Chaomei Chen at Drexel University, this software enables the analysis of research data from various perspectives, including authors, institutions, countries/regions, references, and keywords (33). CiteSpace presents analysis results in intuitive graphical forms, enabling researchers to comprehend complex research information and discover valuable knowledge (34). At the same time, CiteSpace emphasizes identifying key points in a specific field or its evolving trends, with a particular focus on turning points and critical junctures, facilitating a comprehensive analysis of the development within that domain.

The objective of this study is to investigate the hotspots and development trends in the mechanisms of exercise intervention in T2D over the past decade. Utilizing CiteSpace to construct a scientific knowledge map allows for a comprehensive analysis of the current status and trends in this field. This study provides new insights for basic research and clinical prevention and treatment in exercise intervention for T2D.

## 2 Materials and methods

### 2.1 Search strategy

From January 1, 2013, to December 31, 2022, the publications analyzed in this study were retrieved from the Web of Science Core Collection (WoSCC). This database was selected for its esteemed reputation as a comprehensive and authoritative source for scientometric analysis and scientific document visualization (35). Its systematic data-collection approach has been widely recognized and employed in numerous studies (33, 36, 37). All searches were conducted and downloaded on a single day, specifically April 12, 2023, to minimize potential bias resulting from daily database updates. The search terms applied were “exercise,” “type 2 diabetes,” and “mechanism.” For a comprehensive understanding of the search strategy, refer to **Table 1**.

**Table 1:** Search strategy.

Set	Search query
#1	TS=(type 2 diabetes) OR TS=(Diabetes Mellitus, Noninsulin-Dependent) OR TS=(Diabetes Mellitus, Ketosis-Resistant) OR TS=(Diabetes Mellitus, Ketosis Resistant) OR TS=(Ketosis-Resistant Diabetes Mellitus) OR TS=(Diabetes Mellitus, Non Insulin Dependent) OR TS=(Diabetes Mellitus, Non-Insulin-Dependent) OR TS=(Non-Insulin-Dependent Diabetes Mellitus) OR TS=(Diabetes Mellitus, Stable) OR TS=(Stable Diabetes Mellitus) OR TS=(Diabetes Mellitus, Type II) OR TS=(NIDDM) OR TS=(Diabetes Mellitus, Noninsulin Dependent) OR TS=(Diabetes Mellitus, Maturity-Onset) OR TS=(Diabetes Mellitus, Maturity Onset) OR TS=(Maturity-Onset Diabetes Mellitus) OR TS=(Maturity Onset Diabetes Mellitus) OR TS=(MODY) OR TS=(Diabetes Mellitus, Slow-Onset) OR TS=(Diabetes Mellitus, Slow Onset) OR TS=(Slow-Onset Diabetes Mellitus) OR TS=(Type 2 Diabetes Mellitus) OR TS=(Noninsulin-Dependent Diabetes Mellitus) OR TS=(Noninsulin Dependent Diabetes Mellitus) OR TS=(Maturity-Onset Diabetes) OR TS=(Diabetes, Maturity-Onset) OR TS=(Maturity Onset Diabetes) OR TS=(Type 2 Diabetes) OR TS=(Diabetes, Type 2) OR TS=(Diabetes Mellitus, Adult-Onset) OR TS=(Adult-Onset Diabetes Mellitus) OR TS=(Diabetes Mellitus, Adult Onset)
#2	TS=(Exercises) OR TS=(Physical Activity) OR TS=(Activities, Physical) OR TS=(Activity, Physical) OR TS=(Physical Activities) OR TS=(Exercise, Physical) OR TS=(Exercises, Physical) OR TS=(Physical Exercise) OR TS=(Physical Exercises) OR TS=(Acute Exercise) OR TS=(Acute Exercises) OR TS=(Exercise, Acute) OR TS=(Exercises, Acute) OR TS=(Exercise, Isometric) OR TS=(Exercises, Isometric) OR TS=(Isometric Exercises) OR TS=(Isometric Exercise) OR TS=(Exercise, Aerobic) OR TS=(Aerobic Exercise) OR TS=(Aerobic Exercises) OR TS=(Exercises, Aerobic) OR TS=(Exercise Training) OR TS=(Exercise Trainings) OR TS=(Training, Exercise) OR TS=(Trainings, Exercise)
#3	(#1) AND (#2)
#4	[TS=(mechanism)] AND (#3)

### 2.2 Inclusion and exclusion criteria

Inclusion criteria: Original articles or reviews published in peer-reviewed journals that focus on the mechanisms of exercise intervention in T2D.

Exclusion criteria: 1) Conference abstracts or errata; 2) Unpublished articles; 3) Duplicate publications; 4) Irrelevant articles.

We carefully reviewed the titles and abstracts of the articles, excluding those related to type 1 diabetes, those without diabetes, and those without exercise intervention. We included animal and human studies related to exercise intervention in T2D, encompassing the following types: clinical trial, meta-analysis, randomized controlled trial, review, and systematic review.

### 2.3 Bibliometric and visualization analysis

**Figure 1:**
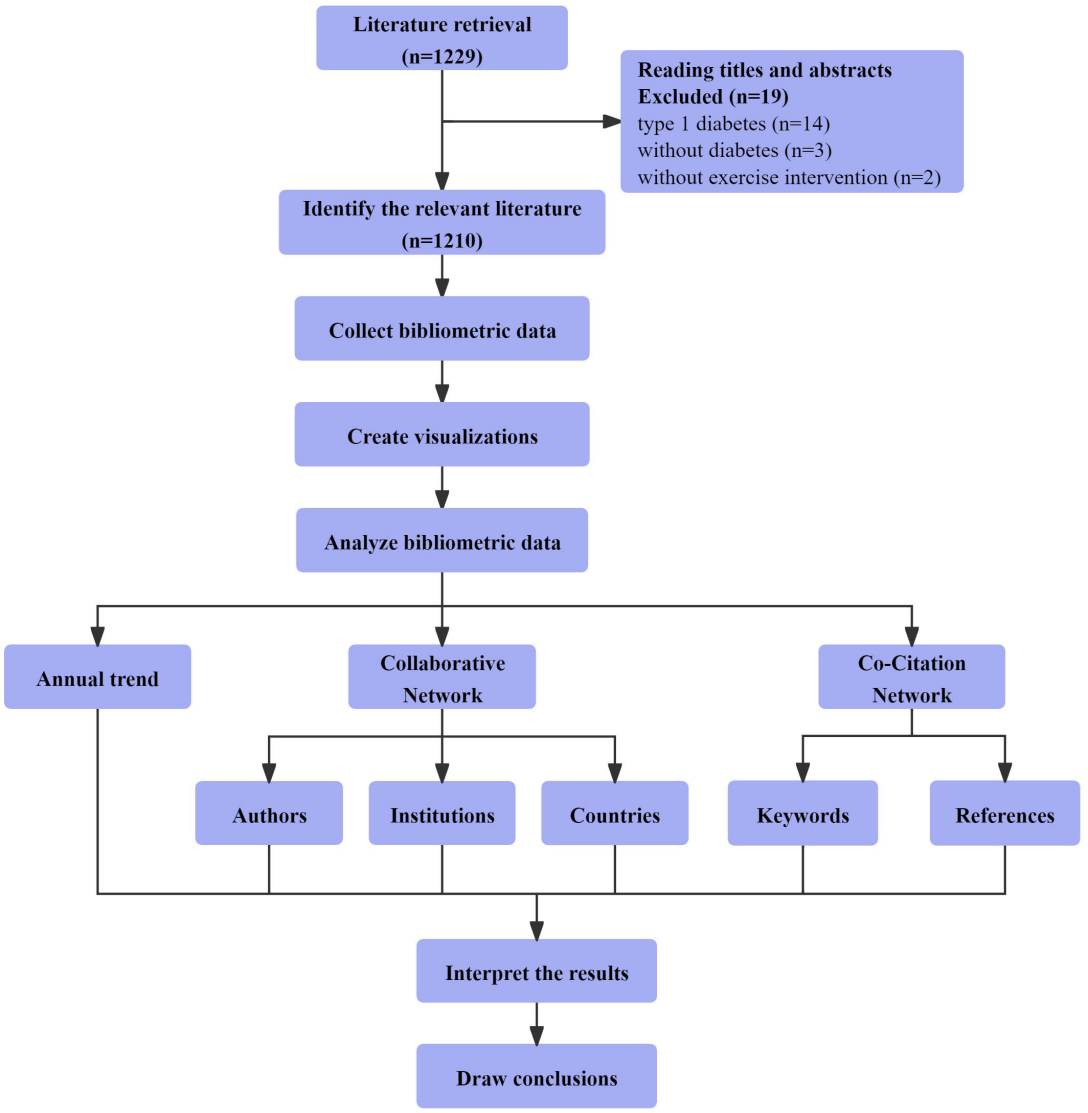
Bibliometric and visualization analysis framework of the mechanisms of exercise intervention in T2D research.

All relevant records meeting the inclusion and exclusion criteria, comprising titles, authors, abstracts, keywords, and references, were exported, converted into plain text files, and labeled as “download_XXX.txt” files. These files were subsequently imported into CiteSpace6.1.R6 for analysis. **Figure 1** visually depicts the 1210 selected papers resulting from scrupulous screening and organization of the retrieved articles. We constructed visual knowledge maps following the key procedural steps of CiteSpace, which include time slicing, thresholding, modeling, pruning, merging, and mapping (38). The settings used for CiteSpace were as follows: link retaining factor (LRF = 3.0), e for top N (e = 1), period (2013–2022), slice length (1), look back years (LBY = 5), links (strength: cosine, scope: within slices), selection criteria (g-index: k = 25), and minimum duration (MD = 1) (37). CiteSpace integrates crucial concepts such as burst detection, betweenness centrality, and diverse networks to visualize the research landscape, hotspots, and frontiers (38). In different maps generated, nodes signify authors, institutions, countries, or keywords. The node size reflects their occurrence or citation frequency, while the node color represents the year of occurrence or citation (34).

In CiteSpace, nodes exhibiting a betweenness centrality surpassing 0.1 are encircled by a purple ring, marking them as pivotal points often acknowledged as hotspots or crucial junctures in the respective field (33, 39). The betweenness centrality metric serves as a network analysis indicator, signifying the intermediary function of nodes within a network (32). The links connecting nodes illustrate associations between two nodes, with an increased number of lines denoting heightened collaboration (37). Line thickness signifies collaboration intensity, whereas thicker lines indicate more substantial cooperation (33). Graph colors denote the distribution across years, transitioning from red to purple, corresponding to the years 2022 to 2013, respectively. Annual research hotspots can be identified by observing the color distribution.

## 3 Results

### 3.1 Annual growth trend

**Figure 2:**
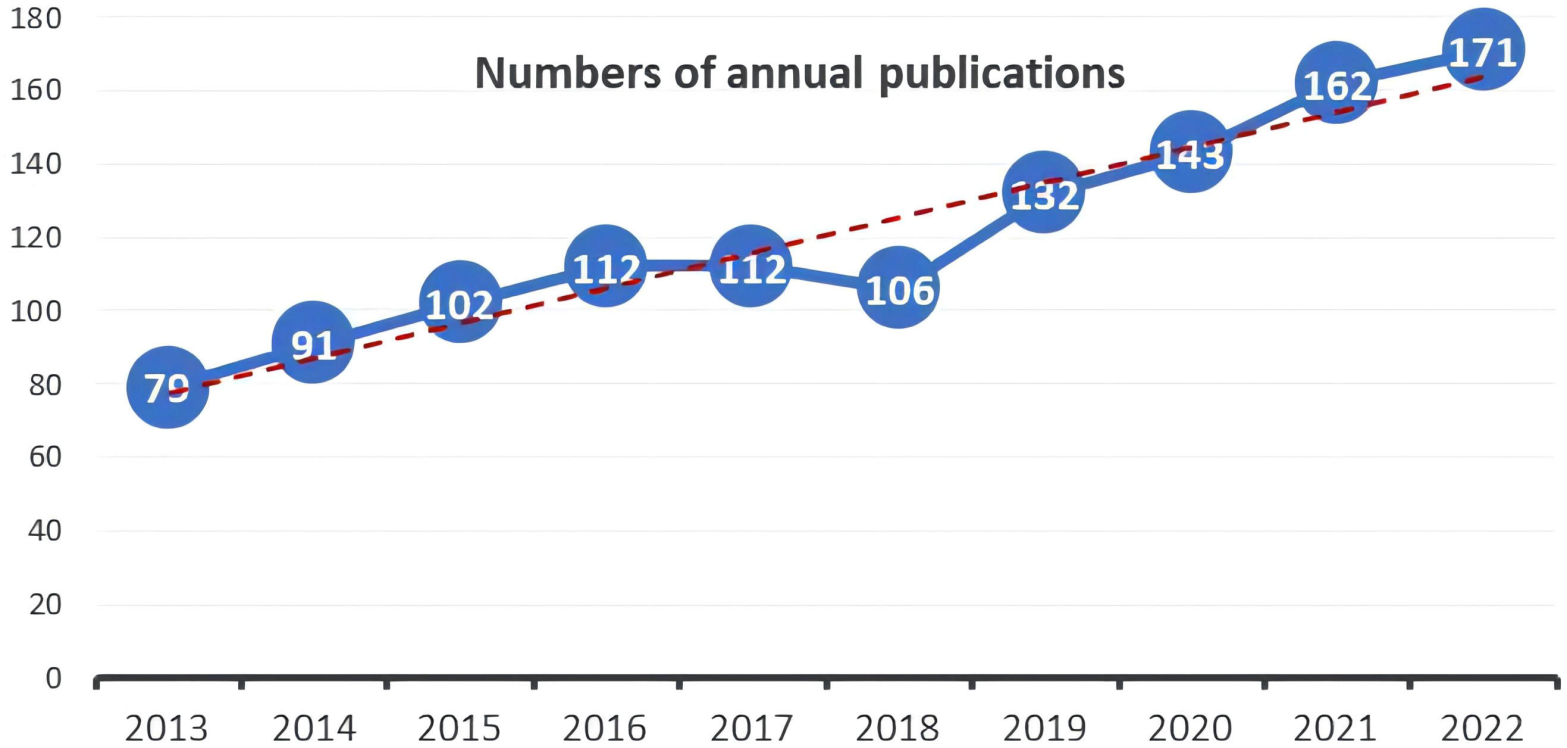
Annual trend of publications.

Out of the 1210 documents analyzed, 772 (63.8%) were articles, while 438 (36.2%) were reviews. The distribution of publication numbers by year, depicted in **Figure 2**, illustrates a consistent upward trend, with a minor dip in 2018. However, there was a significant increase in publications post-2019, indicating an ongoing evolution in the field. In 2013, only 79 publications related to this field were released, while over the subsequent decade, this number has doubled.

### 3.2 Authors, institutions, and national collaborative network

#### 3.2.1 Authors collaborative network

In CiteSpace, the Authors Collaborative Network denotes the collaborative relationships among authors formed through citation connections in academic literature. CiteSpace constructs this network by examining instances where authors co-occur in the literature. When two or more authors are found in the same document, the system identifies a collaborative relationship and establishes the corresponding link in the collaborative network. This network facilitates researchers in discerning the authors who collaborate in a particular field.

**Figure 3:**
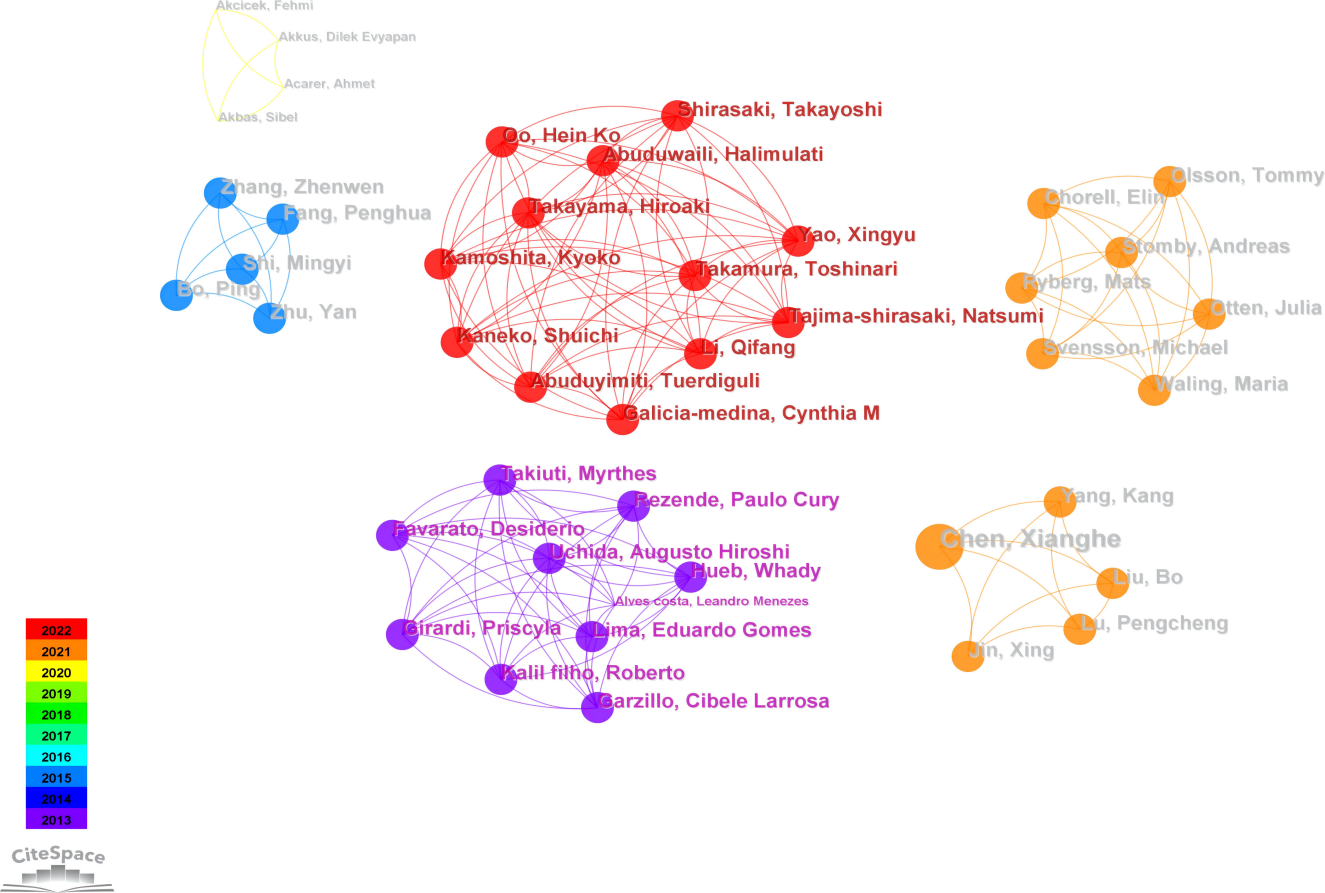
Authors collaboration network graph.

**Figure 3** illustrates an author collaboration network comprising 336 nodes and 264 links, clarifying collaborative relationships among prolific authors. **Table 2** provides information on the top five authors based on publication volume. Most publications are attributed to Professor John P. Kirwan at Case Western Reserve University, with six relevant papers published in the past decade. Professor Kirwan’s team identified growth differentiation factor 15 (GDF15) as a signaling protein generated during skeletal muscle contraction. GDF15 enhances glucose-stimulated insulin secretion (GSIS) by activating the insulin secretion pathway in β-cell, playing a crucial role in regulating glucose metabolism during exercise (40). Professor Kirwan collaborates closely with the second-highest contributor, Professor Steven K. Malin, from the Department of Kinesiology and Health at Rutgers University. Professor Malin is dedicated to determining optimal exercise and diet prescriptions to reduce the risk of chronic diseases (22, 41, 42). His focus includes identifying how each “dose” of exercise impacts cardiovascular and metabolic insulin sensitivity in individuals at risk for diabetes (43).

**Table 2:** Top 5 authors by publication volume from 2013 to 2022.

Author	Publication Volume	Country	Institution	Publication Year	Citations
Kirwan, John P	6	USA	Case Western Reserve University	2014	129
Malin, Steven K	5	USA	Rutgers University	2014	304
Pedersen, Bente Klarlund	4	Denmark	University of Copenhagen	2016	320
Leoni de sousa, Ricardo Augusto	4	Brazil	Federal University of the Valleys of Jequitinhonha and Mucuri	2021	37
Al-Hasani, Hadi	4	Germany	Heinrich Heine University Düsseldorf	2016	264

The third-highest contributor in terms of publication volume is Professor Bente Klarlund Pedersen from the University of Copenhagen. Her research centers on the impact of exercise on physical health, particularly its effects on physiological processes such as metabolism, the immune system, and inflammation (44–46). Professor Pedersen has achieved noteworthy results in exercise and chronic diseases (47–49). Additionally, she advocates combining different types of exercise to enhance health and prevent chronic diseases. Her research indicates that both supervised high-intensity interval endurance training and resistance training effectively reduce cardiac fat in individuals with abdominal obesity, with resistance training specifically targeting pericardial adipose tissue (50). Furthermore, her studies demonstrate that high-intensity interval training (HIIT) can offer similar or superior benefits in physical fitness, body composition, and glycemic control compared to endurance training, making HIIT a time-efficient option for managing T2D (51).

The largest connected component showcases the collaboration network of Professor Toshinari Takamura’s team at Kanazawa University in Japan. The team rediscovered Selenoprotein P (SeP) as a hepatokine overproduced in T2D, leading to insulin and exercise resistance (52). As early as 2017, the team demonstrated that the anti-oxidative hepatokine SeP induces exercise resistance through its muscle receptor, low-density lipoprotein receptor-related protein 1 (LRP1) (53). Subsequently, the team delved further into the mechanisms of exercise resistance in individuals with T2D.

Utilizing the collaborative network established by the authors facilitates the identification of influential contributors in specific fields. It also allows for the exploration of potential research teams or collaborative partnerships. This functionality is essential for understanding the dynamics, trends, and patterns of research collaboration within the academic community. According to the results, John P. Kirwan, Steven K. Malin, Bente Klarlund Pedersen have made significant contributions to the field of exercise intervention for T2D. However, a stable core network of author collaborations has not yet been formed, the betweenness centrality among authors appears modest, suggesting restricted collaboration in this research domain and highlighting the necessity for future efforts to promote more robust and extensive collaborations.

#### 3.2.2 Institutions collaborative network

**Figure 4:**
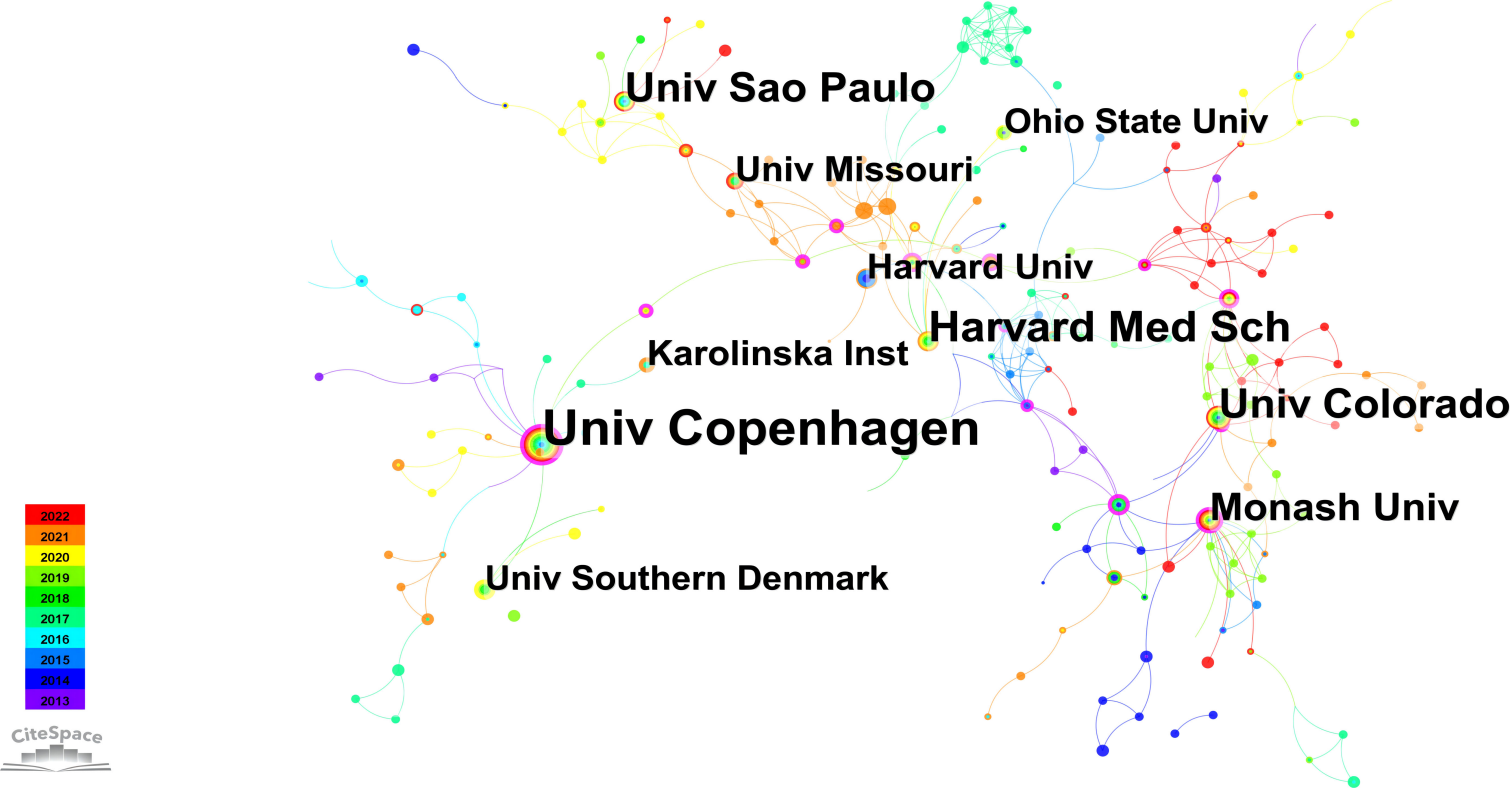
Institutions collaboration network graph.

**Figure 4** depicts an institutional network graph comprising 348 nodes and 558 links, with the top ten prolific institutions marked in the graph. In addition, **Table 3** presents the years of their initial related article publications spanning from 2013 to 2022, along with betweenness centrality. The top three institutions in terms of publication volume are the University of Copenhagen, Harvard Medical School, and the University of Colorado. Notably, the University of Copenhagen has the highest publication volume and demonstrates the strongest betweenness centrality among these institutions. Sustaining an annual research output, the University of Copenhagen exhibits a consistent upward trend in publication volume. As evidenced by the results, the University of Copenhagen has showcased remarkable research achievements in this field. Examining the previously discussed collaborative network, it is evident that Bente Klarlund Pedersen, affiliating with this institution, enjoys a strong reputation for her contributions to improving metabolic diseases through exercise.

**Table 3:** Top 10 institutions by publication volume from 2013 to 2022.

Rank	Publication Volume	Centrality	Publication Year	Institution
1	39	0.33	2013	Univ Copenhagen
2	23	0.09	2016	Harvard Med Sch
3	20	0.04	2013	Univ Colorado
4	19	0.03	2013	Univ Sao Paulo
5	16	0.14	2014	Monash Univ
6	12	0.03	2016	Karolinska Inst
7	12	0.01	2015	Univ Missouri
8	12	0.06	2014	Harvard Univ
9	11	0	2014	Ohio State Univ
10	11	0.01	2018	Univ Southern Denmark

#### 3.2.3 National collaborative network

**Figure 5:**
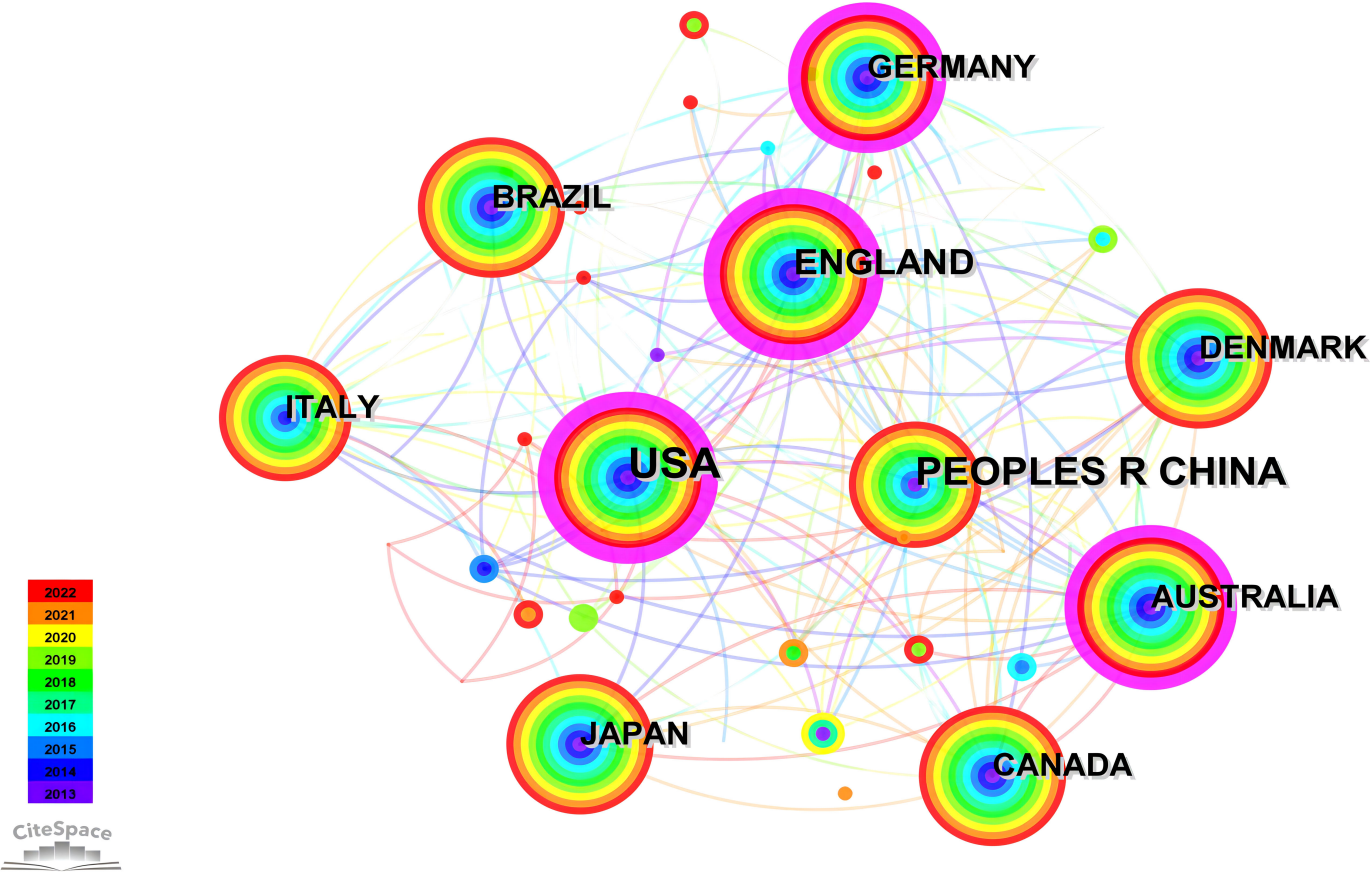
National collaboration network graph.

The network graph for national collaboration comprises 80 nodes and 438 links. We analyzed 1,210 articles from 80 different countries. The findings revealed that the top three countries were USA (n = 382, 31.57%), China (n = 154, 12.73%), and England (n = 111, 9.17%), accounting for over 50% of the total publications, as outlined in **Table 4**. Furthermore, **Figure 5** illustrates collaborative relationships among countries, with node size representing the number of published documents. Interconnections between nodes signify collaborations between countries. Notably, the United States demonstrated significantly higher total link strength than other nations, indicating more substantial collaborations with other countries. The volume of publications in China ranks second only to that of the United States. However, when considering measures such as centrality in comparison with other countries, it appears relatively lower. This suggests that there is room for closer collaboration between China and international counterparts. Strengthening partnerships with institutions from other countries is essential for future endeavors.

**Table 4:** Top 10 countries by publication volume from 2013 to 2022.

Rank	Publication Volume	Centrality	Publication year	Country
1	382	0.38	2013	USA
2	154	0.05	2013	PEOPLES R CHINA
3	111	0.23	2013	ENGLAND
4	85	0.16	2013	GERMANY
5	80	0.13	2013	AUSTRALIA
6	73	0.09	2013	ITALY
7	67	0.04	2013	CANADA
8	66	0.02	2013	BRAZIL
9	63	0	2013	JAPAN
10	57	0.04	2013	DENMARK

### 3.3 Co-citation network

The co-citation network in CiteSpace serves as a network model for examining mutual citation relationships among academic publications. In this network model, nodes signify distinct publications, and links denote instances where these publications are co-cited simultaneously by others. Constructing such a network proves valuable in revealing the interrelations among publications. Co-citation networks are commonly used to discern pivotal publications and thematic clusters and comprehend research focal points within academic disciplines.

#### 3.3.1 Co-citation network of references

Co-cited references denote citations found in two or more publications concurrently. The formation of a co-citation relationship occurs when a third publication references two other publications jointly (54). This interconnection positions co-cited references as a significant knowledge repository within a specific academic domain (55). The importance of a scholarly work in that field increases with the frequency of its citations (37).

**Figure 6:**
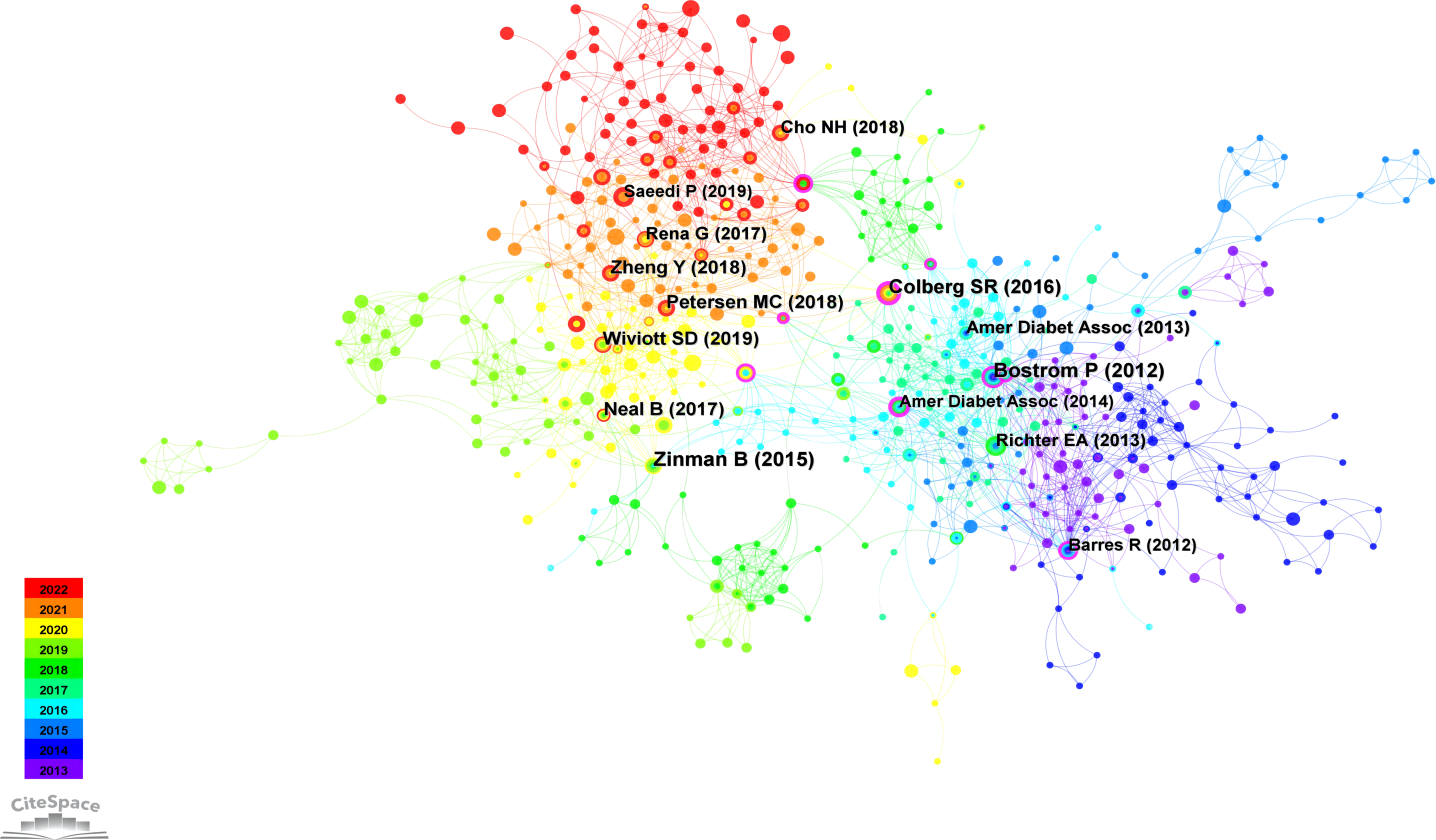
Co-citation network of references.

The findings from the co-citation analysis applied to the cited literature are presented in this section. The co-cited references map reveals 77,107 references cited across 1,210 articles. The co-citation network of references, consisting of 580 nodes and 2,027 edges, illustrates the complex network of co-citation relationships among these references in the academic literature. This observation suggests that, within the subset of 1,210 articles, 580 references were co-cited, resulting in 2,027 instances of simultaneous co-citation. **Figure 6** provides a visual representation of the co-citation network involving frequently cited references, while **Table 5** presents a summary of the top 5 co-cited references.

**Table 5:** Top 5 co-cited references by publication volume from 2013 to 2022.

Publication year	First author	Country/region	Title	Type	Journal	Co-Citations	Centrality	2022 Impact factor
2012	Pontus Boström	USA	A PGC1α-dependent myokine that drives browning of white fat and thermogenesis	Randomized Controlled Trial	Nature	21	0.14	69.504
2015	Bernard Zinman	USA	Empagliflozin, Cardiovascular Outcomes, and Mortality in Type 2 Diabetes	Randomized Controlled Trial	The New England	20	0.09	176.079
2016	Sheri R. Colberg	USA	Physical Activity/Exercise and Diabetes: A Position Statement of the American Diabetes Association	Review	Diabetes Care	18	0.21	17.152
2018	Max C. Petersen	USA	Mechanisms of Insulin Action and Insulin Resistance	Review	Physiological Reviews	16	0.04	46.500
2017	Wiviott SD	USA	Dapagliflozin and Cardiovascular Outcomes in Type 2 Diabetes	Randomized Controlled Trial	The New England Journal of Medicine	16	0.04	176.079

The most extensively cited reference, authored by Bostrom et al. in 2012 and published in Nature, clarified the PGC-1α-dependent myokine irisin. It was revealed that the increased exercise-induced irisin contributes to elevated energy expenditure. Moreover, it is suggested that irisin could have therapeutic potential for human metabolic diseases and other disorders that benefit from exercise (56). Following this, the second most frequently co-cited reference, presented by Zinman et al. in 2015 and published in the New England Journal of Medicine, emphasizes the importance of a comprehensive approach to managing diabetes, encompassing pharmacotherapy and lifestyle modifications such as increased physical activity (57). Colberg’s study in 2016, published in Diabetes Care and ranking third in co-citation count, offers a clinically oriented review along with evidence-based recommendations concerning physical activity and exercise in people with T2D, highlights the significant benefits of physical activity and exercise in reducing blood glucose levels, improving insulin sensitivity, promoting weight reduction, and reveals the importance of personalized exercise programs (12). Occupying the fourth position in co-citation count is Petersen’s study from 2018, published in Physiological Reviews, which underscores the critical role of physical activity and exercise in the management and prevention of diabetes. The research explores the role of insulin and its resistance mechanisms, advocating for comprehensive health management approaches in T2D exercise interventions (58). Lastly, Wiviott’s study from 2017, published in the New England Journal of Medicine and ranking fifth in citation count, provides information on the cardiovascular safety profile of dapagliflozin in individuals with T2D (59).

#### 3.3.2 Co-citation network of keywords

Keywords in CiteSpace play a critical role in defining key terms and facilitating scientific literature’s systematic analysis and visualization of citation networks. These keywords are instrumental in discerning primary themes and pivotal points, enabling researchers to gain detailed insights into developmental trends, research hotspots, and critical contributors within a specific field. The selection of precise and comprehensive keywords ensures the accurate indexing of research articles, as evidenced by previous research (60). Clear and concise presentation of keywords reflects the fundamental themes and perspectives of a scholarly article. As elucidated in earlier studies, it establishes a vital link between diverse research domains (32, 38).

**Figure 7:**
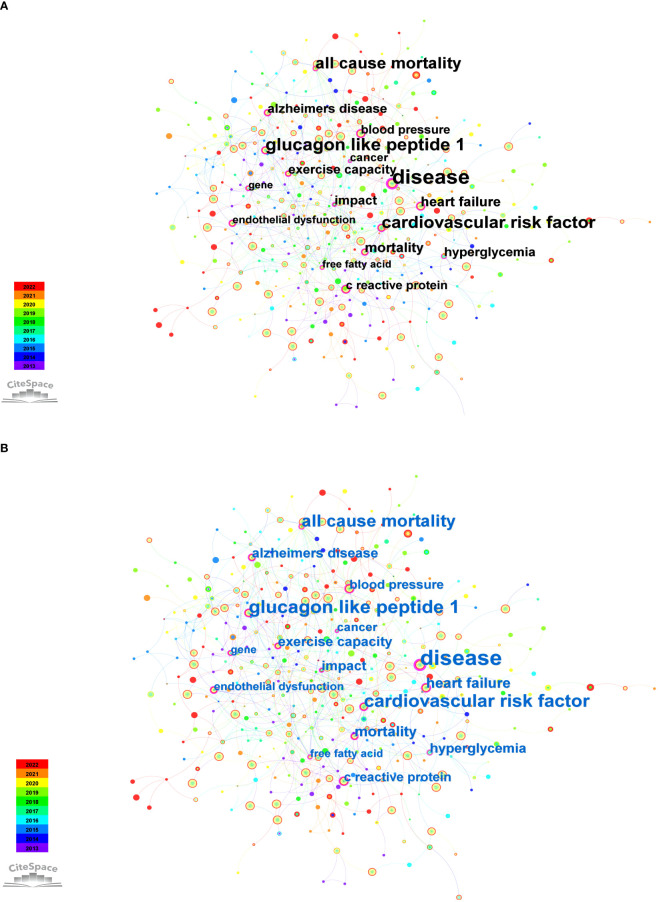
Co-occurrence keywords from 2013 to 2022. **(A)** Top 10 keywords with high frequency. **(B)** Keywords with centrality surpassing 0.1.

In this study, we conducted a time-slicing analysis of annually published papers. **Figure 7A** illustrates the top 10 keywords with high frequency, covering topics such as “type 2 diabetes,” “insulin resistance,” “physical activity,” “skeletal muscle,” “exercise,” “mechanism,” “oxidative stress,” “obesity,” “cardiovascular disease,” and “metabolic syndrome.” Furthermore, we showcased keywords with a centrality exceeding 0.1, as depicted in **Figure 7B**. These keywords, including “disease (0.21),” “glucagon like peptide 1 (0.18),” “cardiovascular risk factor (0.17),” “all cause mortality (0.16),” “heart failure (0.14),” “mortality (0.14),” “Alzheimer’s disease (0.13),” “exercise capacity (0.13),” “hyperglycemia (0.13),” “impact (0.13),” “C-reactive protein (0.12),” “blood pressure (0.12),” “endothelial dysfunction (0.11),” “free fatty acid (0.11),” “cancer (0.11),” “glycemic control (0.1),” “molecular mechanism (0.1),” “diet-induced obesity (0.1),” “induced insulin resistance (0.1),” and “gene (0.1)” illuminate the current research focal points and trends within the field.

##### 3.3.2.1 Keyword network and clustering

**Figure 8:**
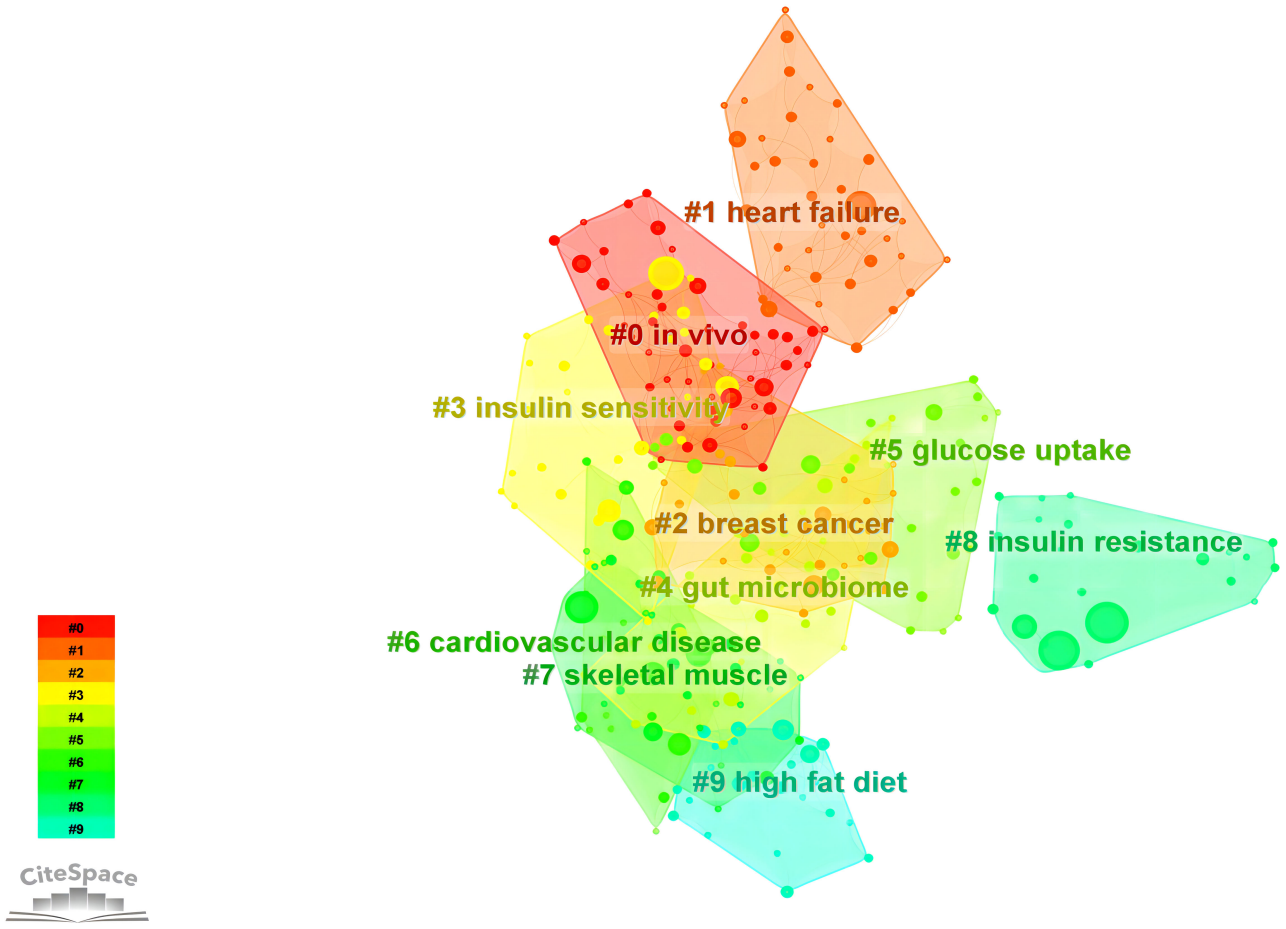
Ten keyword clusters were identified by utilizing the LLR statistic.

Cluster analysis represents a machine learning technique designed explicitly for grouping observed entities, such as literature, keywords, or samples, within a given dataset based on their similarities or distances, commonly denoted as “clusters” (61). The assessment of cluster efficacy frequently relies on utilizing the silhouette coefficient, with a value exceeding 0.7 generally indicating proficient and persuasive clustering outcomes (62). Log-likelihood ratio (LLR) clustering, on the other hand, constitutes a statistically grounded textual clustering approach that computes the likelihood of vocabulary occurrences in a collection of documents (63). We identified ten keyword clusters using the LLR statistic, as illustrated in **Figure 8**. The result demonstrates a robust clustering effect, as evidenced by silhouette coefficients exceeding 0.7 for all clusters (**Table 6**). The elevated silhouette values about the keywords within the clustering process further substantiate the reliability and significance of the obtained results.

**Table 6:** The largest 10 clusters of keywords.

Cluster ID	Size	Silhouette	Mean (Year)	Label (LLR)
0	49	0.904	2015	*in vivo*
1	38	0.855	2016	heart failure
2	31	0.847	2014	breast cancer
3	31	0.842	2018	insulin sensitivity
4	29	0.829	2016	gut microbiome
5	28	0.828	2015	glucose uptake
6	26	0.911	2015	cardiovascular disease
7	25	0.798	2014	skeletal muscle
8	24	0.938	2017	insulin resistance
9	23	0.914	2015	high-fat diet

Based on the findings of keyword clustering, it can be concluded that scholarly research in recent years has placed growing emphasis on integrating fundamental scientific principles with clinical translation. This focus particularly highlights the widespread application of omics technologies *in vivo* experiments, enabling a meticulous molecular exploration to understand the mechanism of exercise’s impact on improving insulin resistance (64–67). Additionally, exercise-induced cytokines exhibit anti-inflammatory effects, contributing to the mitigation of insulin resistance in individuals with T2D and improving glucose tolerance (44).

Moreover, examinations of keyword clustering have revealed a correlation between T2D and the incidence of breast cancer (68, 69). The metabolic dysregulation state in the body fosters the development of breast cancer (70, 71), with low adiponectin levels in obese patients emerging as a significant risk factor for breast cancer occurrence (72).

##### 3.3.2.2 Keyword citation burst

**Figure 9:**
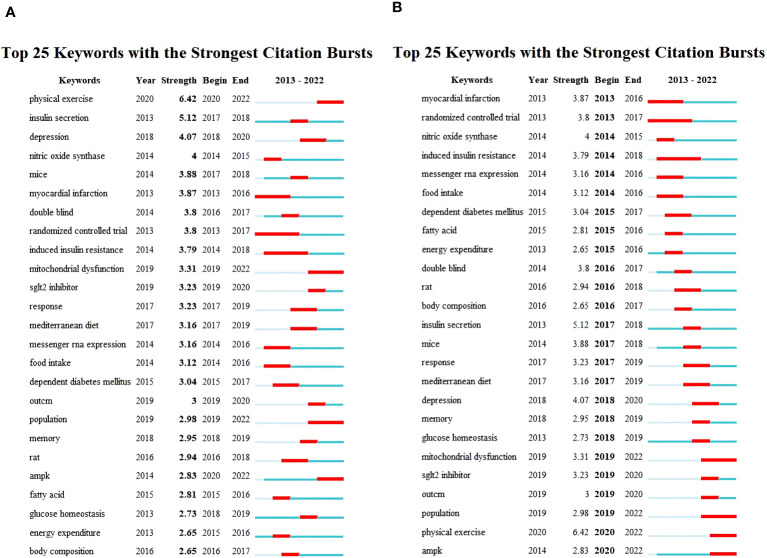
Top 25 keywords with strongest citation bursts. **(A)** Arranged in descending order based on burst strength. **(B)** Arranged in chronological order according to the appearance of bursts.

**Figure 9** illustrates the top 25 keywords with the strongest citation bursts, presenting their initial appearance time, burst strength, begin and end time of burst. **Figure 9A** is arranged in descending order of burst strength, while **Figure 9B** is arranged chronologically based on the occurrence of bursts. The blue line segment shows the overall timeframe, while the red line segment highlights the precise period during which citations were most frequently referenced. The term “physical exercise” demonstrated the most substantial citation burst, attaining a burst strength of 6.42 from 2020 to 2022. Subsequently, “insulin secretion” reached a burst strength of 5.12 in 2017-2018, while “depression” exhibited a burst strength of 4.07 from 2018 to 2020. Other keywords exhibiting notable burst strength encompass nitric oxide synthase (strength: 4; period: 2014-2015), mice (3.88; 2017-2018), myocardial infarction (3.87; 2013-2016), double-blind (3.8; 2016-2017), randomized controlled trial (3.8; 2013-2017), induced insulin resistance (3.79; 2014-2018), mitochondrial dysfunction (3.31; 2019-2022), SGLT2 inhibitor (3.23; 2019-2020), response (3.23; 2017-2019), Mediterranean diet (3.16; 2017-2019), messenger RNA expression (3.14; 2014-2016), food intake (3.12; 2014-2016), dependent diabetes mellitus (3.04; 2015-2017), outcome (3, 2019–2020), population (2.98; 2019-2022), memory (2.95; 2018-2019), rat (2.94; 2016-2018), AMPK (2.83; 2020-2022), fatty acid (2.81; 2015-2016), glucose homeostasis (2.73; 2018-2019), energy expenditure (2.65; 2015-2016), and body composition (2.65; 2016-2017). Furthermore, this graphical representation delineates that AMPK, mitochondrial dysfunction, nitric oxide synthase, SGLT2 inhibitor, messenger RNA expression, exercise outcomes, and exercise response have surfaced as focal points of research attention in recent years. Future research may focus on these aspects.

## 4 Discussion

### 4.1 Collaborative analysis of scientific research networks

The collaborative networks in scientific research constitutes a pivotal aspect of the CiteSpace software. Through the analysis of research collaboration networks generated by CiteSpace, researchers can acquire a comprehensive understanding of collaborative patterns within the research domain, formulate effective collaboration strategies, and identify the research trend and hotspots.

A total of 1210 relevant publications spanning from 2013 to 2022 were retrieved. Over the past decade, an increasing number of researchers have focused on the mechanisms of exercise intervention in T2D, which could be attributed to the increasing prevalence of diabetes over the past decade and the growing recognition of the importance of exercise. Aerobic and resistance exercises have been widely acknowledged as crucial methods for improving glycemic control in T2D individuals. However, the optimal exercise prescription remains unclear, and the mechanisms by which different types of exercise improve T2D are still under investigation. These factors have collectively driven more focused and intensive research in this field, leading to a significant rise in publication volume. The outcome of these research efforts has led to the emergence of a prolific group of authors, including John P. Kirwan, Steven K. Malin, Bente Klarlund Pedersen, Ricardo Augusto Leoni de Sousa, and Hadi Al-Hasani. Analysis of national collaboration networks reveals that the United States holds a prominent position in this research domain, displaying a robust intermediary role in international collaborations. Research institutions and universities in China have demonstrated noteworthy research outcomes in recent years, marked by a substantial increase in published papers. However, compared to the United States, Chinese scholars operate within a relatively closed research circle in this field, with limited international collaboration. Institutional-level analysis indicates that over the past decade, among the top 10 institutions with the highest publication volume, the University of Copenhagen in Denmark claims the foremost position with the highest publication count (39 papers) and the highest intermediary centrality (0.33), signifying substantial research influence in this field.

Highly cited authors and literature primarily focus on the epidemiology and characteristics of T2D, emphasizing that comprehensive health management is a crucial determinant in improving diabetes symptoms. The results of keyword clustering indicate that terms such as “*in vivo*,” “heart failure,” “breast cancer,” “insulin sensitivity,” “gut microbiome,” “glucose uptake,” “cardiovascular disease,” “skeletal muscle,” “insulin resistance,” and “high-fat diet” reflect research trends over the past decade. Recent additions such as “pancreatic β-cell,” “exerkines,” and “microRNA” highlight current focal points in mechanistic research. Additionally, terms like “circadian clock,” “HIIT,” and “responsiveness” shed light on hot topics within the realm of exercise interventions for T2D. Subsequent discussion and analysis will be structured around these aspects.

### 4.2 Biological effects and molecular mechanisms related to exercise

#### 4.2.1 Enhancement of mass and function in pancreatic β-cell

T2D is characterized by a progressive decline in pancreatic β-cell function and insulin resistance (73, 74), where pancreatic β-cell plays a central role in the pathogenesis of this complex multi-organ disease. Current approaches to diabetes treatment focus on strategies aimed at enhancing β-cell signal transduction and function. Exercise has emerged as an effective intervention to improve pancreatic β-cell function (73, 75), particularly in the early stages of the disease, leading to increased insulin secretion and reduced blood glucose levels. However, the complete exploration of the impact of physical exercise on β-cell remains an ongoing focus in current research.

Various forms of exercise have been demonstrated to effectively enhance the functionality of pancreatic β-cell. A study in diabetic fatty rats indicates that a 13-week swimming training regimen effectively enhances β-cell insulin signal transduction and secretion. This improvement is associated with increased cell surface levels of GLUT2, intracellular glucose kinase, and insulin vesicles within β-cell (76). Additionally, another research showcases that a ten-week regimen of resistance exercise training in healthy mice enhances glucose-stimulated insulin secretion and improves β-cell function (77). After an 8-week period of low-volume HIIT in individuals with T2D, there was a notable improvement in overall blood glucose control and pancreatic β-cell function (78).

Numerous studies have examined two distinct exercise modalities: moderate- and vigorous-intensity exercise. Multiple research findings indicate moderate and vigorous exercise improves β-cell function, although through distinct mechanisms (79, 80). The specific mechanisms responsible for the positive impact of exercise on β-cell in T2D patients remain unclear. Future investigations should explore critical regulatory factors influencing exercise-induced enhancements in both β-cell mass and function.

#### 4.2.2 The role of exerkines in metabolic regulation

Current research indicates that bioactive molecules activated by physical activity, referred to as exerkines, may play a crucial role in regulating metabolic balance, thereby reducing the risk of metabolic disorders. Exerkines are identified as signaling molecules released in response to acute or chronic exercise, exerting their effects through endocrine, paracrine, and autocrine pathways (27), with potential implications in improving cardiovascular and metabolic functions. Exerkine research primarily focuses on myokines (49), hepatokines, and adipokines (81).

Interleukin-6 (IL-6) stands as one of the earliest-discovered exerkines. In 2000, Pedersen et al. proposed that actively contracting skeletal muscles are a source of IL-6, exerting its effects in a hormone-like manner (82). Following this, IL-6 has been identified as a myokine (83, 84), demonstrating the capability to augment basal glucose uptake and facilitate the translocation of the glucose transporter GLUT4 (85). Additionally, IL-6 can induce the production of anti-inflammatory cytokines, such as interleukin-1 receptor agonist (IL-1ra) and interleukin-10 (IL-10) (86), while inhibiting the production of tumor necrosis factor-α (TNF-α) (87), thereby manifesting anti-inflammatory effects.

Various exerkines have been extensively studied, including irisin, myostatin, SeP, fetuin-A, fibroblast growth factor 21 (FGF21), interleukin-15 (IL-15), follistatin (FST), β-aminoisobutyric acid (BAIBA), angiopoietin-like 4 (ANGPTL4), chitinase 3-like protein 1 (CHI3L1), cysteine-rich angiogenic inducer 61 (CYR61), and connective tissue growth factor (CTGF) (88–93).

Consequently, these bioactive molecules may represent viable therapeutic avenues for conditions such as cardiovascular diseases, T2D, and obesity.

#### 4.2.3 miRNAs as early indicators in T2D

The concept of the “developmental origins of health and disease” (DOHaD) is based on epidemiologic observations, linking the health status and disease risk in late childhood and adulthood to early-life environmental conditions. This concept involves research on organ development and epigenetic mechanisms, suggesting that chronic non-communicable diseases result from the combined influence of genetic, environmental, and behavioral factors (94).

MicroRNAs (miRNAs), short regulatory RNAs involved in post-transcriptional gene regulation (95), play a fundamental role in the post-transcriptional regulation of gene expression, and participate in various physiopathological processes (96). The secretion of miRNAs into the blood can reveal subtle alterations in key metabolic organs. Changes in the miRNAs profile in the blood are associated with the occurrence of T2D (97). Research has found that in both healthy individuals and those with diabetes, different types of exercise training can regulate the expression of miRNAs (98, 99). Notably, some miRNAs exhibit opposite expression trends in T2D and post-exercise, such as miR-15a, miR-96, miR-192, and miR-532 (100). This suggests that exercise can improve T2D by modulating the expression of miRNAs.

A specific miRNA of interest is miR-126. Its primary involvement extends to angiogenesis, anti-inflammatory responses, and apoptosis, contributing to cardiovascular protection. Multiple clinical studies demonstrate a downregulation of circulating miR-126 levels in patients with T2D (101). Diverse forms of exercise have been identified as regulators of miRNA expression (100). Aerobic exercise has been shown to induce an elevation in circulating levels of miR-126 in mice and rats with metabolic disorders.

Hence, miRNAs can function as an early biomarker to assess the susceptibility to metabolic diseases in later life, assisting in identifying populations prone to obesity and metabolic disorders. This discovery presents new prospects for personalized, precision healthcare and exercise interventions.

### 4.3 Exercise intervention approaches

#### 4.3.1 Exercise type and intensity

Exercise interventions play a crucial role in T2D management by enhancing insulin sensitivity, optimizing blood glucose control, and mitigating the risk of complications. Previous investigations suggest that aerobic exercise, resistance exercise, and HIIT benefit individuals with T2D.

Aerobic exercises, such as brisk walking, jogging, swimming, and cycling, are deemed preferable for patients with T2D due to their substantial enhancement of cardiovascular fitness and insulin sensitivity (102–104). Resistance training, encompassing weightlifting and resistance bands, improves muscle strength, muscle mass, and insulin sensitivity. Combining aerobic exercise with resistance training has become the most common and effective intervention for T2D. Researchers are increasingly focusing on HIIT compared to these two intervention approaches. HIIT, characterized by short bursts of high-intensity exercise followed by moderate or low-intensity recovery, has demonstrated effectiveness in enhancing insulin sensitivity, glycemic control, pancreatic β-cell function, and reducing abdominal fat mass (78).

However, the precise influence of distinct exercise modalities on the pathophysiological mechanisms of T2D still needs to be clarified. Moreover, prevailing studies predominantly emphasize the short-term ramifications of exercise interventions, primarily focusing on parameters such as blood glucose regulation and insulin sensitivity. More attention should be directed towards comprehending the prolonged consequences of exercise interventions, particularly concerning the complications associated with T2D.

In summation, exercise interventions emerge as a vital component in managing T2D. Future research endeavors should formulate and implement personalized exercise intervention programs to achieve more productive disease management and prevention. Furthermore, the temporal aspects of exercise may play a pivotal role in the treatment strategy for T2D.

#### 4.3.2 Exercise timing

The keyword analysis found particular significance in the term “circadian clock.” Several critical physiological processes in the human body, including blood pressure regulation, temperature maintenance, and immune function, follow a day-night rhythm pattern (105). The biological clock plays a pivotal role in influencing essential processes such as the cell cycle, redox homeostasis, inflammation, and metabolism (106). The circadian clock comprises the central and peripheral clocks, with the skeletal muscle clock falling under the peripheral category. Notably, the skeletal muscle clock demonstrates heightened sensitivity to physical activity, resulting in varied transcriptional and metabolic outputs at different times of the day (107). A normal circadian rhythm is crucial for stabilizing internal glucose metabolism (108). Disruptions in circadian rhythm are closely linked to the onset of T2D (108, 109). Factors such as sleep, light exposure, and diet, which are regulated by the circadian rhythm, can significantly impact the development of T2D (108). This raises the question of whether exercising at different times of the day might have varying effects on improving T2D. This finding prompts consideration of whether exercise timing could be considered a viable therapeutic strategy for T2D (110). Nevertheless, there are relatively few studies on this topic, and the intervention durations are often short. A 12-week randomized controlled trial found that exercising three times per week in the morning compared to the evening did not lead to significant differences in glycemic control or alterations in circadian rhythm (111). Look AHEAD trial shows that afternoon bout-related moderate-to-vigorous physical activity (bMVPA) is associated with improved blood glucose control in adults with T2D, particularly within the initial 12 months of intervention (112). Future research should include more intervention studies focusing on exercise timing to gain a deeper understanding of its impact on the circadian rhythm in T2D. This would be crucial for developing precise exercise prescriptions.

### 4.4 Variability in exercise response

Exercise is universally acknowledged and recommended as a fundamental strategy for diabetes and obesity prevention (113, 114). However, its benefits are not universal, as some individuals experience exercise resistance (115, 116). Extensive research reveals that standardized exercise interventions do not uniformly yield improvements in metabolic and cardiorespiratory endpoints for all individuals, showcasing substantial variability in responses (117–119). The correlation between genetics and adaptability to exercise was established as early as the 1980s (120, 121). In recent years, increasing studies have delved into the intrinsic mechanisms of exercise response heterogeneity, prompting a shift in exercise interventions from standardized paradigms to precision approaches (116, 122).

Emerging research highlights that SeP, an antioxidative hepatokine, contributes to exercise resistance in T2D. Upregulated in T2D, SeP leads to increased insulin resistance. This effect occurs through SeP binding to the LRP1 receptor on the surface of skeletal muscle cells, initiating the production of endogenous antioxidants like glutathione peroxidase 1 (GPx1). These antioxidants reduce reactive oxygen species (ROS) during exercise, resulting in decreased AMPK phosphorylation and lower PGC-1α levels, minimizing the promotion of health benefits through exercise (53).

The term “non-response to exercise” refers to the absence of significant changes in specific parameters assessed after training. These parameters encompass fitness, cardiovascular events, muscle mass, metabolic risk status, lipid metabolism, insulin resistance, and glucose homeostasis, and they all require correlation with specific endpoint indicators (116). The efficacy of individual responses to exercise intervention is a relative concept. The absence of a response in one organ or physiological function does not preclude other organs or functions from benefiting from exercise intervention.

In a study of individuals with T2D undergoing ten months of moderate-intensity aerobic training, 35% of patients were classified as “non-responders” due to decreased phosphocreatine (PCr) recovery rate, increased insulin sensitivity, and elevated HbA1c levels. However, both “responders” and “non-responders” demonstrated an approximately 12% improvement in peak oxygen uptake (VO2peak) (123). These findings contrast with traditional distinctions between two different responder groups (124).

The primary reasons for variations in individual responses to exercise lie in the synergistic or antagonistic effects of multiple genetic variations (116). Therefore, it is essential to consider, within the T2D population, which type, frequency, and intensity of exercise yield optimal outcomes for individuals with diverse baseline indicators. This consideration contributes to future research and the formulation of precise exercise prescriptions.

## 5 Conclusions

This study used CiteSpace to analyze the hotspots and frontier issues in research on the mechanisms of exercise intervention in T2D. The results indicate that changes in biological effects and molecular mechanisms, such as improvement in pancreatic β-cell function, exerkines, and epigenetic mechanisms, have become the focus of research. Emerging research is concentrating on exercise response heterogeneity, circadian rhythm regulation, transcription factors, neurotrophic factors, and mitochondrial function. Future research directions should explore the interactions between different mechanisms and elucidate appropriate modes, dosages, and intensities of exercise for precise intervention in T2D.

However, the study has several limitations. Firstly, the analysis is based on available data from published literature, which may not encompass all relevant studies due to publication bias. Secondly, the study focuses on a limited number of databases, potentially overlooking significant research from other sources. Thirdly, the heterogeneity in study designs and participant characteristics across the included studies may affect the generalizability of the findings. Future research should aim to address these limitations by including a broader range of databases and considering unpublished data to minimize bias. Additionally, there is a need for more longitudinal studies to understand the long-term effects of exercise interventions on T2D. Finally, future studies should investigate the personalized exercise prescriptions tailored to individual genetic profiles and baseline characteristics to optimize therapeutic outcomes.

## Author contributions

YJ: Conceptualization, Methodology, Writing – original draft. KW: Conceptualization, Methodology, Writing – original draft. CL: Writing – original draft. WC: Writing – review & editing. RW: Supervision, Funding acquisition, Writing – review & editing.
